# Synthesis of the reported structure of piperazirum using a nitro-Mannich reaction as the key stereochemical determining step

**DOI:** 10.3762/bjoc.9.200

**Published:** 2013-08-23

**Authors:** James C Anderson, Andreas S Kalogirou, Michael J Porter, Graham J Tizzard

**Affiliations:** 1 Department of Chemistry, University College London, 20 Gordon Street, London, WC1H 0AJ, UK; 2 National Crystallography Service, School of Chemistry, University of Southampton, Southampton, SO17 1BJ, UK

**Keywords:** alkaloid, aza-Henry, natural products, nitro-Mannich, piperazinone, stereoselective synthesis

## Abstract

Piperazirum, isolated from *Arum palaestinum* Boiss, was originally assigned as *r*-3,*c*-5-diisobutyl-*c*-6-isopropylpiperazin-2-one. The reported structure was synthesised diastereoselectively using a key nitro-Mannich reaction to set up the C5/C6 relative stereochemistry. The structure was unambiguously assigned by single crystal X-ray diffraction but the spectroscopic data did not match those reported for the natural product. The structure of the natural product must therefore be revised.

## Introduction

**Scheme 1:**
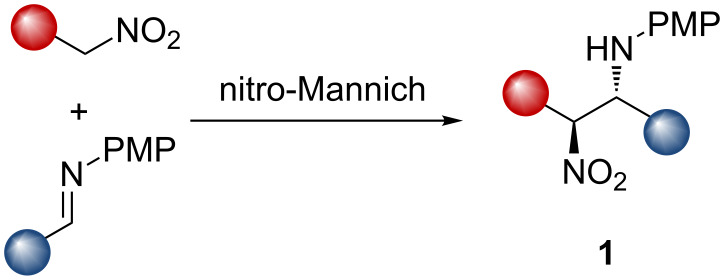
Schematic nitro-Mannich reaction.

The nitro-Mannich reaction (or aza-Henry reaction) has been developed to a standard where the product β-nitroamines **1** are now privileged building blocks. In part this is due to the complementary synthetic flexibility available from the two different nitrogen atom oxidation states (Scheme 1) [1]. They have been used to synthesise many nitrogen-containing functional groups including α-amino carbonyls [2–3], peptidomimetics [4], natural products [5–10] and many heterocyclic small molecules [11–24] of importance to drug discovery. Enantioselective reactions have been controlled by asymmetric metal-centred Lewis acids; chiral hydrogen bond donors, in particular by the use of asymmetric thiourea organocatalysts, chiral Brønsted acids, phase-transfer catalysts and Brønsted base catalysts [3,15,25–39]. A large part of our own work in developing the nitro-Mannich reaction was to demonstrate the preparation of stereodefined 1,2-diamines [40–45].

**Scheme 2:**
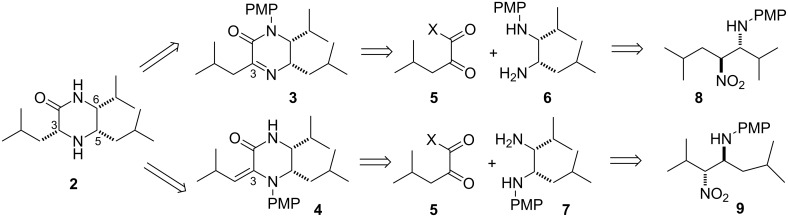
Retrosynthetic analysis of piperazirum.

As part of a programme aimed at using these 1,2-diamines as building blocks in target synthesis we focused on the synthesis of a novel bioactive alkaloid, piperazirum (**2**, Scheme 2), which to our knowledge had not been previously synthesised. Piperazirum was isolated from the leaf extract of *Arum palaestinum* Boiss and was shown to possess significant cytotoxicity against cultured tumor cell lines in vitro [46]. Its chemical structure and relative stereochemistry were elucidated by high resolution mass spectrometry, infrared, 1D and 2D NMR spectroscopy [46]. Retrosynthetically we envisaged that the C-3 stereocentre could be set up by hydrogenation from the less hindered face of α-iminolactam **3** or α,β-unsaturated lactam **4** (Scheme 2). These heterocycles could be derived from a common α-keto acid derivative **5** and either diamine **6** or **7**, that could in turn be prepared from β-nitroamine **8** or **9**. Each of the β-nitroamines could be prepared enantioselectively by using our previously reported methodology [28,43,45] and would allow elucidation of the absolute stereochemistry of piperazirum (**2**).

## Results and Discussion

**Scheme 3:**
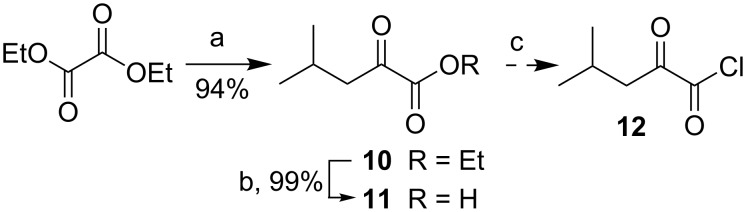
(a) iBuMgCl, Et_2_O, −78 °C; (b) KOH, EtOH/H_2_O, 100 °C; (c) (COCl)_2_.

The common α-keto acid derivative **5** was easily prepared from a Grignard reaction of isobutylmagnesium chloride with diethyl oxalate to give α-keto ester **10** in 94% yield (Scheme 3) [47]. Saponification of **10** with KOH provided α-keto acid **11** in excellent yield [48], and the corresponding acid chloride **12** was prepared in situ by treatment with oxalyl chloride [49].

**Scheme 4:**
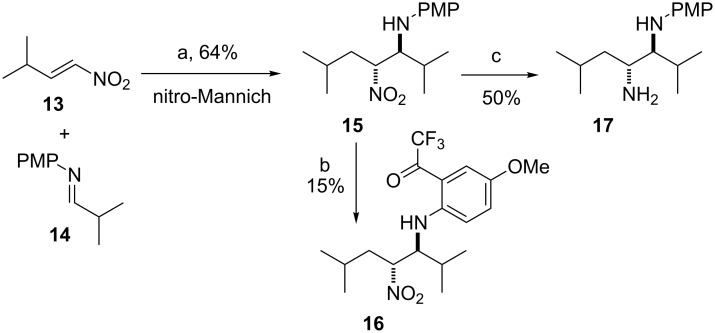
(a) Li(Et_3_BH), THF, rt then **14**, CF_3_CO_2_H, −78 °C, dr 70:30; (b) (CF_3_CO)_2_O, Py, CH_2_Cl_2_, 0 °C to rt; (c) Zn, 6 M HCl, EtOAc/EtOH, rt.

For the synthesis of β-nitroamine **8** we decided to make use of the reductive nitro-Mannich reaction as the starting nitroalkene **13** is readily available from a Henry reaction [50], imine **14** from the condensation of *p*-anisidine and isobutyraldehyde, and the process can easily be made asymmetric [45]. Conjugate addition of hydride to **13** and subsequent trapping of the nitronate anion with freshly prepared imine **14** in THF gave β-nitroamine **15** in 64% conversion and a dr of 70:30 (Scheme 4). Quite frequently β-nitroamines are unstable and susceptible to retroaddition [43–44]. Formation of the corresponding trifluoroacetamide derivative confers stability and allows them to be purified. Using previously developed conditions to protect the amine in situ using (CF_3_CO)_2_O gave only β-nitroamine **16** as a single diastereoisomer in a low 15% yield. These results are consistent with the poor conversions and dr, as well as resistance to trifluoroacetamide protection, we have observed before from imines derived from α-branched aldehydes such as cyclohexanecarbaldehyde [44].

In cases where trifluoroacetylation fails, it is quite common to purify the β-nitroamine by rapid column chromatography, quickly followed by reduction to give the corresponding 1,2-diamine. In this case rapid purification of **15** followed by reduction with Zn/HCl gave the 1,2-diamine **17** as a single diastereoisomer in 50% yield (Scheme 4).

With diamine **17** in hand, the reaction with a suitable keto acid derivative was investigated. We presumed that the nitrogen of the primary amine would be the more nucleophilic and hence should react with the more electrophilic carbonyl group – i.e. the ketone – in **10** [51–52]. Attempted formation of **4** by heating a mixture of **10** and **17** in H_2_O at 50 °C gave a complicated mixture of products and attempts under Dean–Stark conditions in toluene with TsOH gave only recovered starting materials [53].

**Scheme 5:**
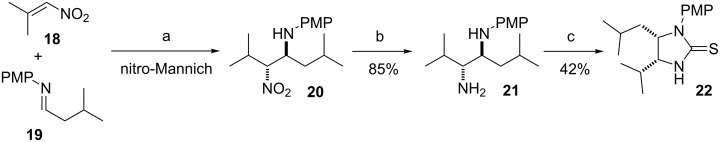
(a) Li(Et_3_BH), CH_2_Cl_2_, rt then **19**, CF_3_CO_2_H, −78 °C, dr >95:5; (b) Zn, 6 M HCl, EtOAc/EtOH, rt, single diastereoisomer; (c) CSCl_2_, aq NaHCO_3_ CH_2_Cl_2_/ MeOH, rt.

In light of this poor result, the alternative route to **2** via β-nitroamine **9** was investigated. A reductive nitro-Mannich reaction between nitroalkene **18** [54] and freshly prepared imine **19** in CH_2_Cl_2_ followed by rapid flash chromatography gave β-nitroamine **20** with complete conversion and dr >95:5 [55]. As before immediate reduction with Zn/HCl gave the PMP-protected diamine **21** in 85% yield as a single diastereoisomer determined by ^1^H NMR (Scheme 5).

From a comprehensive series of examples of the reductive nitro-Mannich reaction, the vast majority of substrates demonstrate *anti*-relative stereochemistry [23,44–45]. More direct proof for **21** was gleaned from the corresponding imidazolidine-2-thione formed by treatment with thiophosgene to give **22** (Scheme 5). In one dimensional nOe studies irradiation of the C*H*NH peak (δ 3.70, 1H, dd, *J* = 8.4, 5.4 Hz) caused a 3.65% enhancement of the C*H*N peak (δ 4.31, 1H, dt, *J* = 7.9, 5.9 Hz), indicating a *cis*-relative stereochemistry between the two protons, which confirmed the *anti*-relative stereochemistry of **21**. The observed coupling constant between the same two protons was 8.2 Hz (averaged) and was similar to other imidazolidine-2-thiones we have prepared that have been corroborated by single crystal X-ray crystallography [56]. Further stereochemical proof was provided by a single crystal X-ray structure determination of **2**·HCl (vide infra).

**Scheme 6:**
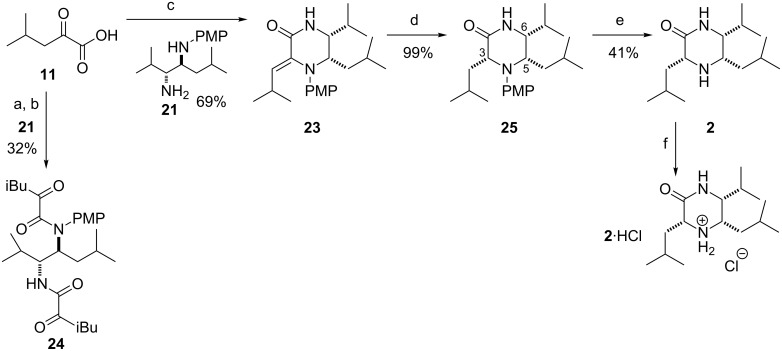
(a) (COCl)_2_, DMF, CH_2_Cl_2_, rt; (b) **21**, Py, DMAP, CH_2_Cl_2_, rt; (c) EDC, HOBt, THF/CH_2_Cl_2_, rt; (d) H_2_ (1 atm), Pd/C, MeOH, rt; (e) CAN, MeCN/H_2_O, 0 °C; (f) 4 M HCl in dioxane, MeCN/Et_2_O.

We presumed again that the primary amine of **21** would be more nucleophilic towards a keto acid derivative **5**. In order to obtain a piperazinone of the desired connectivity (**23**) a keto acid derivative would be required where the carboxylate carbonyl is more reactive than the ketone carbonyl. Two possible such compounds were considered, acid chloride **12** and carboxylic acid **11** treated with a suitable coupling agent. Acid chloride **12** was prepared in situ by treatment of acid **11** with oxalyl chloride (2.00 equiv) and catalytic DMF. Subsequent reaction with diamine **21** in the presence of pyridine (1.20 equiv) and catalytic DMAP over 24 h, according to previously reported reactions for similar keto acids [49], gave only the bis-adduct **24** and none of the desired piperazinone **23**. By contrast, the reaction of carboxylic acid **11** with diamine **21**, in the presence of EDC (1.50 equiv) and 1-hydroxybenzotriazole (1.50 equiv) at rt, gave the desired product **23** in good yield (Scheme 6) [57]. The double bond geometry was assigned as *Z* by NOESY ^1^H NMR and probably results from steric inhibition of resonance which would result in the formation of the *E* iPr group and the planar amide group during formation of **23**.

**Figure 1:**
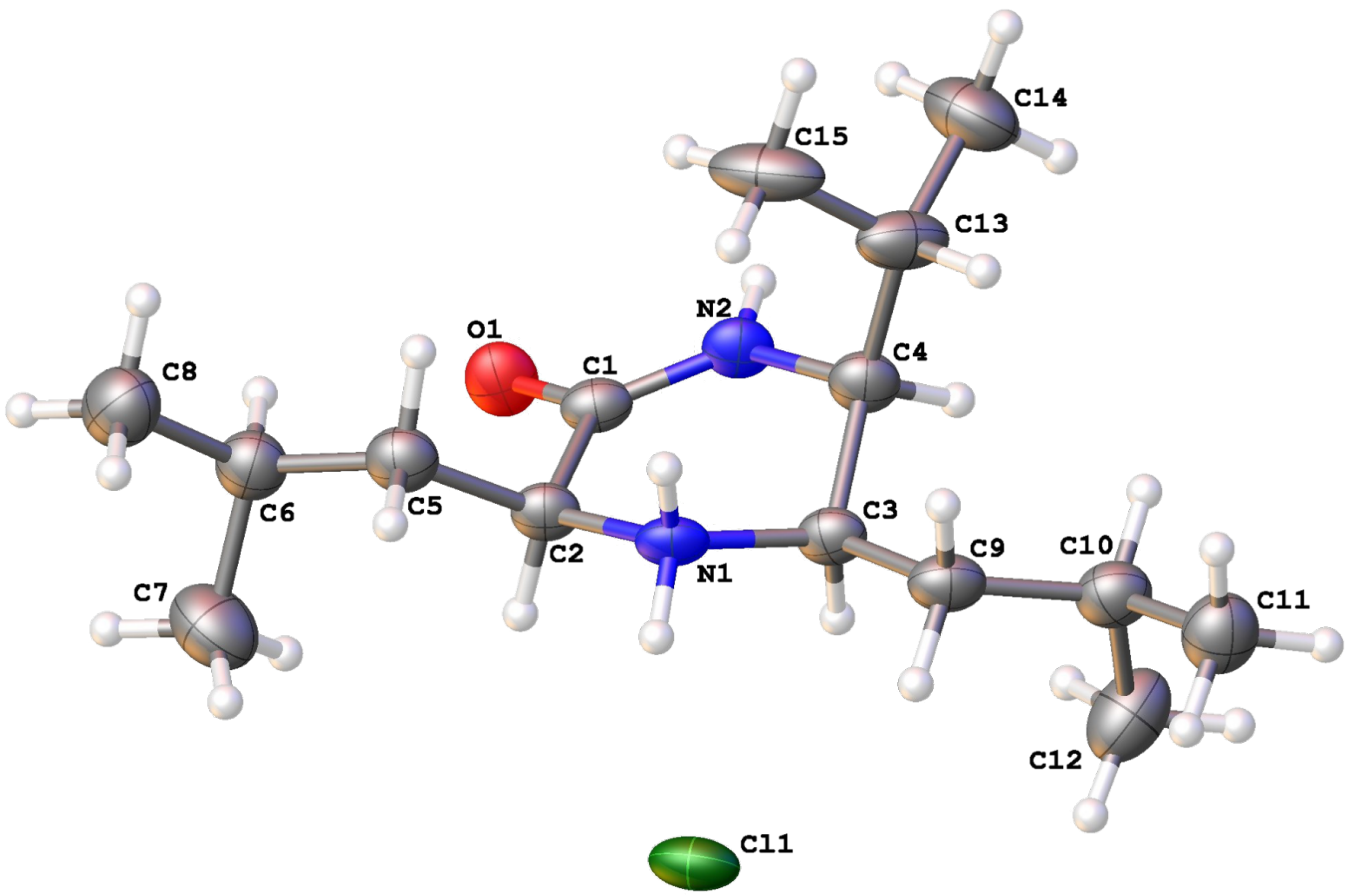
X-ray crystal structure of **2**·HCl.

Reduction of the double bond with hydrogen over palladium on charcoal gave a single diastereoisomer **25** in quantitative yield. In one dimensional nOe studies irradiation of H-3 (δ 4.09, 1H, dd, *J* = 9.9, 4.5 Hz) caused a 0.17% enhancement of the H-6 signal (δ 3.18, 1H, dd, *J* = 10.0, 3.5 Hz) with negligible enhancement of the H-5 signal (δ 3.37, 1H, dt, *J* = 12.4, 3.1 Hz). Irradiation of H-5 caused a 2.90% enhancement of the H-6 signal and a 0.07% enhancement of the H-3 signal. The nOe data tentatively suggested that protons H-3, H-5 and H-6 were all on the same face of the molecule. Deprotection of the PMP group with CAN gave compound **2**. Extensive NMR (COSY, HMBC and NOESY) and analytical data were consistent with the structure drawn (**2**), but the ^1^H and ^13^C NMR did not match that published for piperazirum (Table 1) [46]. The original authors recorded their NMR data for piperazirum in D_2_O [58], but our sample **2** was insoluble. Compound **2** was readily soluble in DMSO-*d*_6_ and CDCl_3_, but neither gave a satisfactory match to the reported NMR data. Preparation of **2**·HCl allowed the recording of NMR spectra in D_2_O, but again the chemical shifts were inconsistent with those reported. Single crystal X-ray structure determination of **2** proved unambiguously the assigned structure obtained from spectroscopic data (Figure 1) [59].

**Table 1:** Comparison of selected ^13^C NMR chemical shifts of piperazirum, **2** and **2**·HCl.

Carbon atom	piperazirum δc (ppm) D_2_O [49]	**2** δc (ppm) DMSO-*d*_6_	**2** δc (ppm) CDCl_3_	**2**·HCl δc (ppm) D_2_O

C-2	175.7	172.5	174.3	169.9
C-3	53.6	56.4	56.9	54.9
C-5	59.7	53.2	53.3	53.9
C-6	60.6	58.1	59.4	56.3
*C*H_2_C-3	40.0	41.5	41.1	38.8
*C*H_2_C-5	24.6	40.2	40.5	36.3

## Conclusion

The rapid synthesis of *r*-3,*c*-5-diisobutyl-*c*-6-isopropylpiperazin-2-one has been achieved using an efficient nitro-Mannich reaction to establish the C-5/C-6 relative stereochemistry which in turn controls the formation of the stereocentre at C-3. Spectrosocpic and single crystal X-ray data have shown that the reported structure for piperazirum is erroneous and that the structure of the natural product needs to be revised. While the reported data point strongly towards piperazirum having the same connectivity as **2**, it is not clear which of the three alternative diastereomers corresponds to the natural product. In view of the lack of natural material, further chemical synthesis, guided by GIAO chemical shift prediction, is currently underway in an effort to elucidate the correct structure for piperazirum. In addition the determination of further biological activity of **2** and its diastereoisomers will be investigated.

## Experimental

Unless otherwise stated, all reactions were carried out under an atmosphere of nitrogen. All glassware was flame dried and allowed to cool under a stream of nitrogen before use. Cooling to 0 °C was effected using an ice–water bath. Cooling to temperatures below 0 °C was effected using dry ice–acetone mixtures. Reactions were monitored by thin layer chromatography (TLC) using 0.25 mm silica precoated plastic plates with fluorescent indicator. Sheets were visualised using ultraviolet light (254 nm) and/or anisaldehyde or KMnO_4_ solutions, as appropriate. Removal of solvents (in vacuo) was achieved using a water aspirator and rotary evaporators. Flash column chromatography was performed using silica gel 60, 40–63 μm. Commercial solvents and reagents were used as supplied or purified in accordance with standard procedures [60]. Diethyl ether (Et_2_O), tetrahydrofuran (THF), dichloromethane (CH_2_Cl_2_) and toluene (PhMe) were obtained from a solvent tower, where degassed solvent was passed through two columns of activated alumina and a 7 micron filter under 4 bar pressure. All NMR samples were made as dilute solutions of CDCl_3_ unless otherwise stated. All chemical shifts (δ) are reported in parts per million (ppm) relative to residual solvent peaks except in D_2_O where they are relative to dioxane (D_2_O) ^1^H 3.75 ppm and ^13^C 67.2 ppm. Multiplicities for ^1^H coupled signals are denoted as s = singlet, d = doublet, t = triplet, q = quartet, quint = quintet, m = multiplet. Coupling constants (*J*) are reported in Hertz. ^13^C multiplicities were assigned using a DEPT sequence. Where appropriate, HMQC, COSY, HMBC and NOE experiments were carried out to aid assignment. Melting points are uncorrected.

**(*E*)-4-Methoxy-*N*-(3-methylbutylidene)aniline (19):** To a mixture of *p-*anisidine (123 mg, 1.00 mmol) and basic alumina (1.00 g) in CH_2_Cl_2_ (5 mL) at −78 °C was added isovaleraldehyde (107 µL, 1.00 mmol) and the mixture stirred for 1 h, then warmed to rt, filtered and evaporated in vacuo to give crude imine **19** (182 mg, 95%) as a colourless oil that was used immediately without further purification; ^1^H NMR (600 MHz) δ 1.02 (d, *J* = 6.7, 6H, C*H*_3_), 2.04 (sept, *J* = 6.7, 1H, C*H*(CH_3_)_2_), 2.34 (dd, *J* = 7.0, 5.4, 2H, C*H*_2_), 3.80 (s, 1H, OC*H*_3_), 6.87 (app. d, *J* = 8.9, 2H, Ar*H*), 7.02 (app. d, *J* = 8.9, 2H, Ar*H*), 7.86 (t, *J* = 5.4, 1H, N=C*H*); ^13^C NMR (150 MHz) data were in agreement to that reported [61].

(3R*,4S*)-*N*^4^-(4-Methoxyphenyl)-2,6-dimethylheptane-3,4-diamine (21): To a solution of nitroalkene **18** (202 mg, 2.00 mmol), in CH_2_Cl_2_ (12.0 mL) was added Superhydride^®^ (2.20 mL, 1 M in THF, 2.20 mmol) and the mixture stirred for 15 min at rt. The mixture was cooled to −78 °C before the dropwise addition of a solution of freshly prepared imine **19** (564 mg, 4.00 mmol) in CH_2_Cl_2_ (12.0 mL). The reaction was stirred for 10 min before the dropwise addition of a solution of CF_3_CO_2_H (460 µL, 6.00 mmol) in CH_2_Cl_2_ (4.0 mL). The reaction was stirred for 1 h and then quenched with brine (20 mL) at −78 °C, warmed to rt and extracted with Et_2_O (3 × 20 mL). The combined organics were dried (MgSO_4_) and evaporated in vacuo to give crude β-nitroamine **20**, that was purified by column chromatography (petrol ether/Et_2_O 4:1). Subsequent reaction with 6 M HCl (6.60 mL, 40.0 mmol) and Zn dust (1.30 g, 20.0 mmol) and purification by flash column chromatography (CH_2_Cl_2_/MeOH 10:1) gave diamine **21** (452 mg, 85%) as a brown oil; *R*_f_ 0.50 (CH_2_Cl_2_/MeOH 10:1); IR ν_max_ (thin film): 3374 (br, N-H), 2955 (w, C-H), 1618 (w), 1508 (s, C=C), 1465 (m), 1441 (w), 1385 (w), 1366 (w), 1292 (w), 1238 (s), 1179 (w), 1154 (w), 1038 (m), 816 (m), 752 (s) cm^−1^; ^1^H NMR (600 MHz) δ 0.90 (d, *J* = 6.5, 3H, C*H*_3_), 0.92 (d, *J* = 6.7, 3H, C*H*_3_), 0.99 (d, *J* = 6.6, 6H, C*H*_3_), 1.21 (m, 1H, C*H*_2_), 1.35 (m, 1H, C*H*_2_), 1.59 (m, 1H, C*H*(CH_3_)_2_), 1.80 (m, 1H, C*H*(CH_3_)_2_), 2.53 (dd, *J* = 9.1, 2.7, 1H, C*H*NH_2_), 3.52 (m, 1H, C*H*NH), 3.73 (s, 3H, OC*H*_3_), 6.57 (app. d, *J* = 8.9, 2H, Ar*H*), 6.76 (app. d, *J* = 8.9, 2H, Ar*H*); ^13^C NMR (150 MHz) δ 19.6 (*C*H_3_), 20.6 (*C*H_3_), 21.7 (*C*H_3_), 24.1 (*C*H_3_), 24.7 (*C*H(CH_3_)_2_), 31.4 (*C*H(CH_3_)_2_), 37.2 (*C*H_2_), 52.9 (*C*HNH), 55.8 (O*C*H_3_), 59.1 (*C*HNH_2_), 114.4 (*C*H arom.), 115.0 (*C*H arom.), 142.3 (*C*q arom.), 151.6 (*C*q arom.); MS (EI^+^) *m/z*: 264 (M^+^, 5%), 192 (M^+^ − (CH_3_)_2_CHCHNH_2_, 100%); HRMS *m/z*: calcd for C_16_H_28_N_2_O, 264.2196; found, 264.2201.

(5S*,6R*,*Z*)-5-Isobutyl-6-isopropyl-4-(4-methoxyphenyl)-3-(2-methylpropylidene)piperazin-2-one (23): To a solution of diamine **21** (61 mg, 0.23 mmol) in THF (5 mL) was added *N*-(3-dimethylaminopropyl)-*N*′-ethylcarbodiimide hydrochloride (EDC, 67 mg, 0.35 mmol) and 1-hydroxybenzotriazole hydrate (47 mg, 0.35 mmol) followed by a solution of keto acid **11** (30 mg, 0.23 mmol) in CH_2_Cl_2_ (5 mL) at rt. The mixture was stirred for 24 h, then diluted with CH_2_Cl_2_ (20 mL) and washed with brine (10 mL). The combined organic phases were dried (MgSO_4_), evaporated in vacuo and purified by flash column chromatography (petrol ether/EtOAc 7:3) to give piperazinone **23** (57 mg, 69%) as a brown solid; mp 152–153 °C; *R*_f_ 0.30 (petrol ether/EtOAc 7:3); IR ν_max_ (thin film): 3209 (br, N-H), 2956 (w, C-H), 1671 (s, C=C), 1622 (s), 1499 (s), 1464 (m), 1442 (m), 1409 (m), 1384 (m), 1366 (m), 1331 (m), 1283 (m), 1241 (s), 1180 (m), 1153 (m), 1037 (m), 826 (s), 767 (m) cm^−1^; ^1^H NMR (600 MHz) δ 0.68 (d, *J* = 6.6, 3H, C*H*_3_), 0.79 (d, *J* = 6.6, 3H, C*H*_3_), 0.88 (d, *J* = 6.6, 3H, C*H*_3_), 0.93 (d, *J* = 6.5, 3H, C*H*_3_), 0.95 (d, *J* = 6.7, 3H, C*H*_3_), 1.07 (d, *J* = 6.5, 3H, C*H*_3_), 1.07 (m, 1H, C*H*_2_), 1.55 (m, 1H, C*H*_2_), 1.55 (m, 1H, C*H*(CH_3_)_2_), 1.94 (m, 1H, C*H*(CH_3_)_2_), 2.43 (m, 1H, C*H*(CH_3_)_2_), 3.18 (dd, *J* = 10.3, 3.6, 1H, C*H*NH), 3.53 (br. d, *J* = 12.2, 1H, NC*H*CH_2_), 3.76 (s, 3H, OC*H*_3_), 5.89 (br. s, 1H, N*H*), 6.48 (d, *J* = 10.6, 1H, =C*H*), 6.78 (app. d, *J* = 8.8, 2H, Ar*H*), 6.89 (app. d, *J* = 8.8, 2H, Ar*H*); ^13^C NMR (150 MHz) δ 17.9 (*C*H_3_), 19.4 (*C*H_3_), 20.3 (*C*H_3_), 21.5 (*C*H_3_), 22.2 (*C*H_3_), 23.7 (*C*H_3_), 24.1 (*C*H(CH_3_)_2_), 26.4 (*C*H(CH_3_)_2_), 29.0 (*C*H(CH_3_)_2_), 34.4 (*C*H_2_), 55.4 (O*C*H_3_), 58.0 (*C*HNH), 58.8 (N*C*HCH_2_), 114.3 (*C*H arom.), 122.0 (*C*H arom.), 129.7 (CH=*C*q), 140.0 (Cq=*C*H), 143.4 (*C*q arom.), 154.3 (*C*q arom.), 164.3 (*C*=O); MS (EI^+^) *m/z*: 359 (MH^+^, 100%), 343 (12%), 192 (13%); HRMS *m/z*: calcd for C_22_H_35_N_2_O_2_, 359.2693; found, 359.2678.

(3R*,5S*,6R*)-3,5-diisobutyl-6-isopropyl-4-(4-methoxyphenyl)piperazin-2-one (25): To a solution of piperazinone **23** (170 mg, 0.470 mmol) in MeOH (10 mL) was added palladium on carbon (50 mg, 10% by weight, 0.047 mmol) and the mixture was flushed with hydrogen, then stirred under a hydrogen atmosphere (balloon) at rt. After the piperazinone starting material was consumed (TLC, 4 h) the mixture was filtered through celite^®^, washed with CH_2_Cl_2_ (20 mL) and evaporated in vacuo to give crude piperazinone that was purified by flash column chromatography (petrol ether/Me_2_CO 4:1) to give piperazinone **25** (170 mg, 99%) as a colourless oil; *R*_f_ 0.50 (petrol ether/Me_2_CO 4:1); IR ν_max_ (thin film): 3207 (br, N-H), 2954 (m, C-H), 1658 (s, C=O), 1505 (C=C), 1465 (m), 1367 (m), 1242 (s), 1180 (m), 1039 (m), 827 (m), 788 (m), 733 (m) cm^−1^; ^1^H NMR (600 MHz) δ 0.70 (d, *J* = 6.5, 3H, C*H*_3_), 0.77 (d, *J* = 6.7, 3H, C*H*_3_), 0.90 (d, *J* = 6.7, 3H, C*H*_3_), 0.97 (d, *J* = 6.5, 3H, C*H*_3_), 0.98 (d, *J* = 6.8, 3H, C*H*_3_), 1.03 (d, *J* = 6.5, 3H, C*H*_3_), 1.03 (m, 1H, CHCHC*H*_2_), 1.55 (m, 1H, CHCHC*H*_2_), 1.55 (m, 1H, O=CCHC*H*_2_), 1.55 (m, 1H, CHC*H*(CH_3_)_2_), 1.84 (m, 1H, O=CCHC*H*_2_), 1.92 (m, 1H, O=CCHCH_2_C*H*(CH_3_)_2_), 2.06 (m, 1H, CHCHCH_2_C*H*(CH_3_)_2_), 3.18 (dd, *J* = 10.0, 3.5, 1H, NHC*H*), 3.37 (dt, *J* = 12.4, 3.1, 1H, NHCHC*H*N), 3.77 (s, 3H, OC*H*_3_), 4.09 (dd, *J* = 9.9, 4.5, 1H, O=CC*H*), 6.02 (br. s, 1H, N*H*), 6.80 (app. d, *J* = 8.9, 2H, Ar*H*), 6.91 (app. d, *J* = 8.9, 2H, Ar*H*); ^13^C NMR (150 MHz) δ 18.0 (*C*H_3_), 19.5 (*C*H_3_), 21.5 (*C*H_3_), 21.6 (*C*H_3_), 23.3 (*C*H_3_), 23.4 (CHCHCH_2_*C*H(CH_3_)_2_), 23.7 (*C*H_3_), 24.7 (O=CCHCH_2_*C*H(CH_3_)_2_), 29.2 (NHCH*C*H(CH_3_)_2_), 34.7 (*C*H_2_CHCH), 44.0 (*C*H_2_CHC=O), 55.4 (O*C*H_3_), 57.2 (N*C*HC=O), 58.4 (N*C*HCH), 58.5 (NH*C*H), 114.5 (*C*H arom.), 122.6 (*C*H arom.), 146.2 (*C*H arom.), 154.3 (*C*H arom.), 173.9 (*C*=O); MS (EI^+^) *m/z*: 360 (M^+^, 15%), 303 (18%), 192 (100%); HRMS *m/z*: calcd for C_22_H_36_N_2_O_2_, 360.2771; found, 360.2774.

(3R*,5S*,6R*)-3,5-diisobutyl-6-isopropylpiperazin-2-one (2): To a solution of piperazinone **25** (320 mg, 0.880 mmol) in MeCN (10 mL) at 0 °C was added a solution of CAN (2.08 g, 3.52 mmol) in H_2_O (10 mL) dropwise over 3 min. The solution turned from pale yellow to dark orange. The mixture was stirred at 0 °C for 2 h, over which time the solution became light orange. Water (30 mL) was then added and the mixture extracted with EtOAc (3 × 20 mL), washed with saturated aqueous NaHCO_3_ (40 mL), dried (MgSO_4_) and evaporated in vacuo to give crude piperazinone that was purified by flash column chromatography (petrol ether/Me_2_CO 3:2) to give piperazinone **2** (91 mg, 41%) as a brown oil; *R*_f_ 0.53 (petrol ether/Me_2_CO 3:2); IR ν_max_ (thin film): 3209 (w, N-H), 2955 (C-H), 1658 (s, C=O), 1467 (m), 1367 (m), 1165 (w), 918 (w), 722 (w) cm^−1^; ^1^H NMR (600 MHz, CDCl_3_) δ 0.89 (d, *J* = 6.6, 3H, C-3CH_2_CHC*H_3_*), 0.90 (d, *J* = 6.2, 3H, C-5CH_2_CHC*H_3_*), 0.91 (d, *J* = 5.6, 3H, C-6CHC*H_3_*), 0.93 (d, *J* = 6.9, 3H, C-3CH_2_CHC*H_3_*), 0.94 (d, *J* = 6.8, 3H, C-5CH_2_CHC*H_3_*), 0.98 (d, *J* = 6.7, 3H, C-6CHC*H_3_*), 1.30 (ddd, *J* = 13.9, 7.1, 6.5, 1H, C-5C*H*_2_), 1.33 (ddd, *J* = 13.9, 8.2, 5.6, 1H, C-5C*H*_2_), 1.40 (ddd, *J* = 14.2, 10.0, 4.1, 1H, C-3C*H*_2_), 1.65 (nonet, *J* = 6.7, 1H, C-5CH_2_C*H*), 1.74 (m, 1H, C-3CH_2_C*H*), 1.88 (ddd, *J* = 13.7, 10.3, 3.3, 1H, C-3C*H*_2_), 1.91 (hepd, *J* = 6.8, 2.5, 1H, C-6C*H*), 3.06 (dd, *J* = 6.7, 3.6, 1H, C-6*H*), 3.15 (dt, *J* = 7.8, 5.3, 1H, C-5*H*), 3.40 (dd, *J* = 10.2, 3.4, 1H, C-3*H*), 6.22 (brs, 1H, N^1^*H*), N^4^*H* peak is missing; ^13^C NMR (125 MHz, CDCl_3_) δ 17.7 (C-6CH*C*H_3_), 21.0 (C-3CH_2_CH*C*H_3_), 21.5 (C-5CH_2_CH*C*H_3_), 22.4 (C-6CH*C*H_3_), 23.1 and 23.7 (C-3CH_2_CH*C*H_3_/ C-5CH_2_CH*C*H_3_), 24.4 (C-3CH_2_*C*H), 24.8 (C-5CH_2_*C*H), 27.9 (C-6*C*H), 40.5 (C-5*C*H_2_), 41.1 (C-3*C*H_2_), 53.3 (C-5), 57.0 (C-3), 59.4 (C-6), 174.2 (C=O); MS (EI^+^) *m/z*: 254 (M^+^, 30%), 197 (22%), 169 (43%), 154 (31%); HRMS *m/z*: calcd for C_15_H_30_N_2_O, 254.2353; found, 254.2355.

^1^H NMR (600 MHz, DMSO-*d_6_*) δ 0.83–0.89 (m, 18H, 6xMe), 1.25 (m, 3H, C-3C*H*_2_ + 2xC-5C*H*_2_), 1.65 (m, 2H, C-3CH_2_C*H* + C-5CH_2_C*H*), 1.79 (m, 2H, C-3C*H*_2_ + C-6C*H*), 2.90 (brd, *J* = 3.2, 1H, C-6*H*), 3.01 (td, *J* = 7.1, 3.7, 1H, C-5*H*), 3.21 (dd, *J* = 9.3, 3.2, 1H, C-3*H*), 7.63 (brs, 1H, N*H*). Assignments based on above; ^13^C NMR (125 MHz, DMSO-*d_6_*) δ 18.4, 21.5, 22.0, 22.8, 23.0, 23.8, 23.9, 24.2, 27.4, 40.2, 41.5, 53.2, 56.4, 58.1, 172.5.

In a clean vial HCl in dioxane (~1 equiv) was added to a small sample of **2** in MeCN, the mixture layered with Et_2_O and then left in the fridge until colourless blades of **2**·HCl had formed at the interface. mp 121–123 °C. ^1^H NMR (600 MHz, D_2_O) δ 0.89–0.99 (6xMe), 1.52–1.68 (m, 4H), 1.72–1.79 (m, 1H), 1.94–1.99 (m, 2H), 3.50 (dd, *J* = 6.2, 4.3, 1H, C-6*H*), 3.79 (m, 1H, C-5*H*), 4.01 (dd, *J* = 8.3, 5.3, 1H, C-3*H*); ^13^C NMR (150 MHz, D_2_O) δ 18.8, 20.6, 21.3, 22.0, 22.5, 24.2, 24.7, 25.7, 27.4, 36.3, 38.8, 53.9, 54.9, 56.3, 169.9 (C=O).

## Supporting Information

File 1 Further experimental and characterisation data.
