# Supplementary material for: Synthesis of the reported structure of piperazirum using a nitro-Mannich reaction as the key stereochemical determining step
Source: Beilstein J Org Chem. 2013 Aug 23;9:1737–44. doi: 10.3762/bjoc.9.200 (PMC3778392; doi:10.3762/bjoc.9.200)
Supplement: File 1 — Further experimental and characterisation data. [file Beilstein_J_Org_Chem-09-1737-s001.pdf]

Supporting Information

for

Synthesis of the reported structure of piperazirum using a nitro-Mannich reaction as the key stereochemical determining step

James C. Anderson*[1], Andreas S. Kalogirou[1], Michael J. Porter[1] and Graham J. Tizzard[2,§]

Address: [1]Department of Chemistry, University College London, 20 Gordon Street, London, WC1H 0AJ, UK and [2]National Crystallography Service, School of Chemistry, University of Southampton, Southampton, SO17 1BJ, UK

Email: James C. Anderson* - j.c.anderson@ucl.ac.uk; Graham J. Tizzard[§] - gjt1@soton.ac.uk

* Corresponding author

§ Corresponding author for crystallographic results

Further experimental and characterisation data

(*E*)-4-Methoxy-*N*-(2-methylpropylidene)aniline (14): To a mixture of *p*-anisidine (246 mg, 2.00 mmol) and basic alumina (2.00 g) in CH_2Cl_2 (10 mL) at –78 °C was added isobutyraldehyde (182 μL, 2.00 mmol) and the mixture stirred for 1 h, then warmed to rt, filtered and evaporated in vacuo to give crude imine **14** (343 mg, 89% pure by ^{1}H NMR, 86%) as a colourless oil which was used without further purification; IR ν_{max} (thin film) 2962 w (C-H), 2869 w, 1649 m (C=C), 1503 s, 1464 m, 1441 m, 1291 m, 1239 s, 1211 m, 1179 m, 1105 m, 1034 m, 823 m, 759 m cm^{-1}; ^{1}H NMR (600 MHz) δ 1.18 (6H, d, *J* = 6.9, C*H*$_3$), 2.62 (1H, m, C*H*$(CH_3)_2$), 3.80 (3H, s, OC*H*$_3$), 6.87 (2H, app. d, *J* = 8.8, Ar*H*), 7.02 (2H, app. d, *J* = 8.8, Ar*H*), 7.73 (1H, d, *J* = 4.9, =C*H*); ^{13}C NMR (150 MHz) δ 19.2 (*C*H$_3$), 34.7 (*C*H), 55.4 (O*C*H$_3$), 114.1 (*C*H arom.), 121.7 (*C*H arom.), 145.3 (*C*q arom.), 157.7 (*C*q arom.), 169.4 (=*C*H); *m/z* (EI$^+$) 177 (M$^+$, 100%), 162 (M$^+$ − Me, 53%); HRMS: found 177.1144 $C_{11}H_{15}NO$ requires 177.1148.

1-(2-(((3*R,4*S**)-2,6-Dimethyl-4-nitroheptan-3-yl)amino)-5-methoxyphenyl)-2,2,2-trifluoroethanone (16):** To a solution of nitroalkene **13** (115 mg, 1.00 mmol), in THF (6.0 mL) was added Superhydride® (1.10 mL, 1 M in THF, 1.10 mmol) and the mixture stirred for 15 min at rt. The mixture was cooled to −78 °C before the dropwise addition of a solution of freshly prepared imine **14** (345 mg, 2.00 mmol) in THF (6.0 mL). The reaction was stirred for 10 min before the dropwise addition of a solution of CF_3CO_2H (230 μL, 3.00 mmol) in THF (2.0 mL). The reaction was stirred for 1 h and then quenched with brine (10 mL) at −78 °C, warmed to rt and extracted with Et_2O (3 × 10 mL). The combined organics were dried ($MgSO_4$) and evaporated in vacuo to give crude β-nitroamine **15**. A sample was taken for ^{1}H NMR analysis which showed 64% conversion and dr 70:30.

The rest of the crude product was dissolved in CH_2Cl_2 (5 mL), cooled to 0 °C and trifluoroacetic anhydride (550 μL, 4.00 mmol), pyridine (320 μL, 4.00 mmol) added dropwise, the mixture warmed to rt and stirred for 30 min. The mixture was then washed with 2 M aqueous HCl (3 × 10 mL), brine (10 mL), dried ($MgSO_4$) and concentrated in vacuo. Purification by flash column chromatography (petrol ether/Et_2O 9:1) gave **16** (60 mg, 15%) as a single diastereoisomer and an orange solid; mp 99–100 °C; R_f 0.61 (petrol ether/Et_2O 9:1); IR ν_{max} (thin film) 3310 br (N-H), 2964 w (C-H), 1652 m (C=O), 1582 m, 1552 m (C=C), 1525 s (N-O), 1468 m, 1421 w, 1372 m (N-O), 1265 m, 1233 m, 1193 m (C-F), 1148 s (C-F), 1117 m, 1045 m cm^{-1}; ^{1}H NMR (600 MHz) δ 0.89 (3H, d, *J* = 6.7, C*H*$_3$), 0.90 (3H, d, *J* = 6.3, CH_3), 0.91 (3H, d, *J* = 6.6, C*H*$_3$), 1.04 (3H, d, *J* = 6.8, C*H*$_3$), 1.44 (1H, m, C*H*$_2$), 1.51 (1H, m, C*H*CH_3), 1.87 (1H, m, C*H*CH_3), 2.09 (1H, ddd, *J*

= 14.6, 11.7, 3.5, C*H*$_2$), 3.78 (3H, s, OC*H*$_3$), 4.10 (1H, ddd, *J* = 10.5, 7.9, 4.6, C*H*NH), 4.72 (1H, ddd, *J* = 11.7, 7.9, 2.4, C*H*NO$_2$), 6.92 (1H, app. d, *J* = 9.4, Ar*H*), 7.20 (2H, m, Ar*H*), 8.70 (1H, d, *J* = 10.4, N*H*); ^{13}C NMR (150 MHz) δ 16.3 (*C*H$_3$), 20.0 (*C*H$_3$), 20.9 (*C*H$_3$), 23.3 (*C*H$_3$), 25.1 (*C*H(CH$_3$)$_2$), 30.5 (*C*H(CH$_3$)$_2$), 38.4 (*C*H$_2$), 55.7 (O*C*H$_3$), 60.5 (*C*HNH), 88.2 (*C*HNO$_2$), 110.3 (*C*q arom.), 112.5 (q, *J* = 4.6, Ar*C*H), 114.0 (Ar*C*H), 117.2 (q, *J* = 291.0, *C*F$_3$), 128.4 (Ar*C*H), 149.4 (*C*q arom.), 150.1 (*C*q arom.), 180.3 (q, *J* = 33.3, *C*=O); ^{19}F NMR (282 MHz) δ −69.40 (s, C*F*$_3$); *m/z* (EI$^+$) 390 (M$^+$, 15%), 301 (25%), 274 (100%), 258 (36%); HRMS: found 390.1758, $C_{18}H_{25}F_3N_2O_4$ requires 390.1761; Anal. calcd. for $C_{18}H_{25}F_3N_2O_4$: C, 55.38, H, 6.45, N, 7.18. Found C, 55.43, H, 6.51, N, 6.93%.

(3*R,4*S**)-*N*^{3}-(4-Methoxyphenyl)-2,6-dimethylheptane-3,4-diamine (17):** Prepared by an analogous method to **16** using nitroalkene **13** (230 mg, 2.00 mmol), Superhydride® (2.20 mmol), imine **14** (708 mg, 4.00 mmol) and CF_3CO_2H (460 µL, 6.00 mmol) to give crude β-nitroamine **15**, that was purified by column chromatography (petrol ether/Et_2O 4:1). To a solution of crude **15** in EtOH (20 mL) and EtOAc (20 mL) was added an aqueous solution of 6 M HCl (6.60 mL, 40.0 mmol), followed by Zn dust (1.30 g, 20.0 mmol) in 3 portions over 1 h. The mixture was stirred vigorously for 1 h before the solvents were removed in vacuo. The residue was neutralised with saturated aqueous Na_2CO_3, extracted with EtOAc (3 × 20 mL), washed with brine (10 mL), dried ($MgSO_4$) and concentrated in vacuo to leave the crude diamine that was purified by flash column chromatography (CH_2Cl_2/MeOH 20:1) and gave diamine **17** (248 mg, 50%) as a single diastereoisomer and a white solid; mp 86–87 °C; R_f 0.29 (DCM/MeOH 20:1); IR ν_{max} (thin film) 3374 br (N-H), 2954 m (C-H), 1509 s (C=C), 1465 m, 1230 s, 1178 m, 1039 m, 815 m cm^{-1}; ^{1}H NMR (600 MHz) δ 0.91 (12H, m, C*H*$_3$), 1.21 (2H, m, C*H*$_2$), 1.81 (2H, m, C*H*(CH$_3$)$_2$), 1.81 (2H, m, N*H*$_2$), 2.93 (1H, m, C*H*NH$_2$), 3.05 (1H, m, C*H*NH), 3.15 (1H, br. s, N*H*), 3.72 (3H, s, OC*H*$_3$), 6.62 (2H, app. d, *J* = 8.7, Ar*H*), 6.73 (2H, app. d, *J* = 8.5, Ar*H*); ^{13}C NMR (150 MHz) δ 19.1 (*C*H$_3$), 20.6 (*C*H$_3$), 21.2 (*C*H$_3$), 24.3 (*C*H$_3$), 24.6 (*C*H(CH$_3$)$_2$), 31.3 (*C*H(CH$_3$)$_2$), 41.5 (*C*H$_2$), 50.9 (*C*HNH$_2$), 55.7 (O*C*H$_3$), 65.6 (*C*HNH), 113.9 (*C*H arom.), 114.8 (*C*H arom.), 144.2 (*C*q arom.), 151.3 (*C*q arom.); *m/z* (EI$^+$) 264 (M$^+$, 6%), 178 (100%); HRMS: found 264.2196, $C_{16}H_{28}N_2O$ requires 264.2196.

(4*R,5*S**)-5-Isobutyl-4-isopropyl-1-(4-methoxyphenyl)imidazolidine-2-thione (22):** Saturated aqueous $NaHCO_3$ (2.40 mL) and H_2O (2.40 mL) were added to a solution of diamine **21** (130 mg, 0.490 mmol) in CH_2Cl_2 (14 mL) and MeOH (7 mL), stirred for 5 min at rt and then $CSCl_2$ (55 µL, 0.74 mmol) was added and the mixture stirred for 24 h. Water

(20 mL) was then added and the mixture extracted with CH_2Cl_2 (3 × 20 mL), dried ($MgSO_4$) and evaporated in vacuo to give crude imidazolinethione. Purification by flash column chromatography (petrol ether/EtOAc 4:1) gave imidazolinethione **22** (130 mg, 42%) as a white solid; mp 123–124 °C; R_f 0.53 (petrol ether/EtOAc 4:1); IR ν_{max} (thin film) 3200 br (N-H), 2955 m (C-H), 1612 w, 1515 s (C=O), 1485 m (C=C), 1448 s, 1253 s, 1225 m, 1172 m, 1033 m, 840 m, 808 m cm^{-1}; ^{1}H NMR (600 MHz) δ 0.77 (3H, d, *J* = 6.6, C*H*$_3$), 0.79 (3H, d, *J* = 6.6, C*H*$_3$), 0.96 (3H, d, *J* = 6.8, C*H*$_3$), 1.05 (3H, d, *J* = 6.6, C*H*$_3$), 1.35 (1H, m, CH$_2$C*H*(CH$_3$)$_2$), 1.44 (1H, m, C*H*$_2$), 1.49 (1H, m, C*H*$_2$), 1.96 (1H, m, CHC*H*(CH$_3$)$_2$), 3.70 (1H, dd, *J* = 8.4, 5.4, C*H*NH), 3.82 (3H, s, OC*H*$_3$), 4.31 (1H, dt, *J* = 7.9, 5.9, NC*H*CH$_2$), 6.36 (1H, br. s, N*H*), 6.92 (2H, app. d, *J* = 8.9, Ar*H*), 7.22 (2H, app. d, *J* = 8.9, Ar*H*); ^{13}C NMR (150 MHz) δ 19.1 (*C*H$_3$), 20.6 (*C*H$_3$), 21.2 (*C*H$_3$), 24.3 (*C*H$_3$), 24.6 (*C*H(CH$_3$)$_2$), 31.3 (*C*H(CH$_3$)$_2$), 41.5 (*C*H$_2$), 50.9 (*C*HNH), 55.7 (O*C*H$_3$), 65.6 (*C*HNPMP), 113.9 (Ar*C*H), 114.8 (Ar*C*H), 144.2 (Ar*C*q), 151.3 (Ar*C*q); *m/z* (EI$^+$) 264 (M$^+$, 6%), 178 (100%); HRMS: found 264.2196, $C_{17}H_{26}N_2OS$ requires 264.2196; Anal. calcd. for $C_{17}H_{26}N_2OS$: C, 66.62, H, 8.55, N, 9.14. Found C, 66.30, H, 8.60, N, 8.93%.

***N*-((3*R**,4*S**)-2,6-dimethyl-3-(4-methyl-2-oxopentanamido)heptan-4-yl)-*N*-(4-methoxyphenyl)-4-methyl-2-oxopentanamide (24):** To a solution of keto acid **11** (130 mg, 1.00 mmol) in CH_2Cl_2 (2 mL) was added oxalyl chloride (2.00 equiv, 170 µL) and DMF (two drops) and the mixture stirred at rt for 1 h. The solvent and excess oxalyl chloride were then removed in vacuo and a solution of diamine **21** (188 mg, 0.710 mmol) in CH_2Cl_2 (5 mL) was added, followed by pyridine (1.20 equiv, 97 µL) and DMAP (5 mg), and the solution was stirred for 24 h at rt. Water (20 mL) was then added and the mixture extracted with CH_2Cl_2 (3 × 20 mL), dried ($MgSO_4$) and evaporated in vacuo and purification by flash column chromatography (petrol ether/Et_2O 4:1) gave di-amide **24** (111 mg, 32%) as a colourless oil; R_f 0.23 (petrol ether:Et_2O 4:1); IR ν_{max} (thin film) 3352 br (N-H), 2958 m (C-H), 1714 m (C=O), 1684 m (C=O), 1649 s (C=O), 1510 s, 1467 m, 1368 m, 1297 m, 1251 m, 1171 m, 1147 m, 1074 m, 1035 m, 831 m cm^{-1}; ^{1}H NMR (600 MHz) δ 0.71 (3H, d, *J* = 6.6, C*H*$_3$), 0.72 (3H, d, *J* = 6.7, C*H*$_3$), 0.90-1.00 (18H, m, C*H*$_3$), 1.05 (2H, m, CHC*H*$_2$CH), 1.73 (1H, m, C*H*(CH$_3$)$_2$), 1.97 (1H, m, C*H*(CH$_3$)$_2$), 2.12 (2H, m, C*H*(CH$_3$)$_2$), 2.33 (2H, m, C*H*$_2$), 2.72 (2H, m, C*H*$_2$), 3.78 (3H, s, OC*H*$_3$), 3.87 (1H, m, C*H*NH), 4.83 (1H, br. s, C*H*NAr), 6.77 (1H, br. d, *J* = 10.9, N*H*CO), 6.83 (2H, app. d, *J* = 8.7, Ar*H*), 7.03 (2H, app. d, *J* = 8.7, Ar*H*); ^{13}C NMR (150 MHz) δ 16.7 (*C*H$_3$), 20.2 (*C*H$_3$), 21.8 (*C*H$_3$), 22.1 (*C*H$_3$),

22.4 (*C*H_3), 22.5 (*C*H_3), 23.2 (*C*H_3), 23.6 (*C*H_3), 24.5 (*C*H), 24.8 (*C*H)[two CH signals overlapping], 29.2 (CH_2*C*HNAr), 36.4 (CH*C*H_2CH), 45.2 (*C*H_2CO), 49.1 (*C*H_2CO), 55.4 (O*C*H_3), 55.8 (*C*HNHCO), 114.5 (*C*H arom.), 127.3 (*C*q arom.), 131.1 (*C*H arom.), 159.8 (ArN*C*=O), 160.2 (*C*q arom.), 169.3 (NH*C*=O), 198.6 (CH_2*C*=O), 200.2 (CH_2*C*=O); *m/z* (CI^+) 489 (M + H^+, 60%), 471 (M + H^+ − H_2O, 61%), 359 (40%), 93 (100%); HRMS: found 489.3341, $C_{28}H_{45}N_2O_5$ requires 489.3323.
